# Expanding the phenotype of reciprocal 1q21.1 deletions and duplications: a case series

**DOI:** 10.1186/s13052-017-0380-x

**Published:** 2017-07-19

**Authors:** Martina Busè, Helenia C. Cuttaia, Daniela Palazzo, Marcella V. Mazara, Salvatrice A. Lauricella, Michela Malacarne, Mauro Pierluigi, Simona Cavani, Maria Piccione

**Affiliations:** 1 Department of Sciences for Health Promotion and Mother and Child Care “Giuseppe D’Alessandro”, University of Palermo, Palermo, Italy; 2 Laboratory of Medical Cytogenetic, AOOR Villa Sofia-Cervello, Palermo, Italy; 3 Regional Referral Centre for Rare Genetic and Chromosomal Diseases, AOOR Villa Sofia-Cervello, Palermo, Italy; 4 S.C. Laboratory of Human Genetics, E.O. Galliera Hospital, Genoa, Italy

**Keywords:** 1q21.1 deletion, 1q21.1 duplication, Array-CGH, Developmental delay, Dysmorphism

## Abstract

**Background:**

Recurrent reciprocal 1q21.1 deletions and duplications have been associated with variable phenotypes. Phenotypic features described in association with 1q21.1 microdeletions include developmental delay, craniofacial dysmorphism and congenital anomalies. The 1q21.1 reciprocal duplication has been associated with macrocephaly or relative macrocephaly, frontal bossing, hypertelorism, developmental delay, intellectual disability and autism spectrum disorder.

**Methods:**

Our study describes seven patients, who were referred to us for developmental delay/intellectual disability, dysmorphic features and, in some cases, congenital anomalies, in whom we identified 1q21.1 CNVs by array-CGH.

**Results:**

Our data confirm the extreme phenotypic variability associated with 1q21.1 microdeletion and microduplication. We observed common phenotypic features, described in previous studies, but we also described, for the first time, congenital hypothyroidism in association with 1q21.1 deletion and trigonocephaly associated with 1q21.1 duplication.

**Conclusions:**

The aim of this study is to contribute to the definition of the phenotype associated with reciprocal 1q21.1 deletions and duplications.

## Background

In recent years, the introduction of new technologies such as comparative genomic hybridization (CGH) allows for the routine detection of submicroscopic deletions and duplications. Several studies of patients with global developmental delay, intellectual disability and/or congenital malformation of unknow cause have led to the identification of new genomic disorders [1–4].

Recurrent reciprocal 1q21.1 deletions and duplications have been associated with variable phenotypes. Phenotypic features described in association with 1q21.1 microdeletions include developmental delay, craniofacial dysmorphism and congenital anomalies. Developmental delay is usually mild and may involve global or specific areas. Psychiatric and behavioral abnormalities, such as autism spectrum disorders (ASD), schizophrenia and attention deficit hyperactivity disorder (ADHD), are also described in a minority of patients. Dysmorphic features are a common finding; they may include microcephaly (almost 50% of patients), frontal bossing, deep-set eyes, epicanthal folds, large nasal bridge, long philtrum and highly arched palate. Several congenital anomalies may be associated with the deletion: congenital heart disease (CHD), eye abnormalities (microphthalmia, chorioretinal and iris colobomas, strabism, various type of cataracts), skeletal and genitourinary malformations. In some cases, seizures are also described (15%) [5–9]. As it is clear, the phenotype of 1q21.1 microdeletion has a high variability, so it is not possible to define a clinically recognizable syndrome.

Few individuals with 1q21.1 reciprocal duplication have been reported in literature. Most recognizable features are macrocephaly or relative macrocephaly, frontal bossing, hypertelorism, developmental delay, intellectual disability and autism spectrum disorder [6, 7]. Individuals with 1q21.1 copy number variations (CNVs) may also have a normal phenotype.

The 1q21.1 critical region spans approximately 1.35 Mb (from 145 to 146.35 Mb) [6] and includes at least 12 genes, among which *PRKAB2, FMO5, CHD1L, BCL9, ACP6, GJA5, GJA8, GPR89B*. Deletions and duplications can be inherited from a parent in an autosomal dominant manner or occur de novo.

Our study describes seven patients, who were referred to us for developmental delay/intellectual disability, dysmorphic features and, in some cases, congenital anomalies, in whom we identified 1q21.1 CNVs by array-CGH. The aim of this study is to contribute to the definition of the phenotype associated with reciprocal 1q21.1 deletions and duplications.

## Methods

### Clinical reports

All patients were referred to our Regional Referral Centre for Rare Diseases for the presence of developmental delay, intellectual disability, dysmorphic features and/or congenital anomalies. One patient had a prenatal diagnosis, following the identification of anomalies on the obstetric ultrasound. Written informed consents were obtained from all participants.

Patient 1 is a 6-year old girl. She is the third child of healthy, non-consanguineous parents. Her family history is positive for intellectual disability (one brother). She has microcephaly and mild dysmorphic features. Neuropsychiatric evaluation revealed intellectual disability, psychomotor and language delay. EEG (electroencephalogram) and brain CT were reported normal.

Patient 2 is a 8-year old boy, the second child of non-consanguineous parents. He was born at term of gestation by cesarean delivery. He was referred to us because of learning disabilities and encopresis. Physical examination showed growth retardation (all growth parameters lower than 3° percentile) and dysmorphic features: dry hair with abnormal implant, hypotelorism, muscular hypotrophy and bilateral clinodactyly of I, II, IV, V fingers. Brain and pituitary MRI was reported normal.

Patient 3 is the only one with a prenatal diagnosis. His family history is positive for intellectual disability and epilepsy. Obstetric ultrasound, performed at 21 weeks of gestation (WG), showed bilateral cysts of the choroid plexus and a reduction in size in the ossification nucleus of the nose. Because of the presence of this anomalies on ultrasound, an amniocentesis was performed. At birth (40,5 WG) the patient was immediately studied: he had no dysmorphic features and his growth parameters were normal. Brain, heart and abdominal ultrasounds were normal.

Patient 4 is a 2-year old boy, the first child of non-consanguineous parents. His family history is positive for intellectual disability, congenital anomalies (first pregnancy: stillbirth with agenesis of the radius and thumb) and chromosomal abnormalities (trisomy 21). He was born at 39,1 WG by cesarean delivery. He was referred to us because of the presence of severe vesicoureteral reflux (VUR), with hydronephrosis detected prenatally, and dysmorphism. Physical examination showed growth retardation (all growth parameters lower than 3° percentile) and dysmorphic features: prominence of the metopic suture, plagiocephaly, hypotelorism, bilateral clinodactyly of IV and V fingers and toes. Brain and heart ultrasounds were reported normal.

Patient 5 is a 8-year old boy. He is the first child of healthy, non-consanguineous parents. His family history is positive for intellectual disability and microcephaly. He was born at 29,2 WG because of premature rupture of membranes (PROM). Because of his prematurity, he was admitted to neonatal intensive care: he had pneumothorax, respiratory distress and retinopathy of prematurity (ROP). When the patient came to our attention, he had intellectual disability, spastic tetraparesis, strabism and craniofacial dysmorphism: microcephaly, protruding ears, prominent nasal bridge, short philtrum, micrognathia and spaced teeth. Brain MRI showed periventricular leukomalacia, polymicrogyria and dilatation of lateral ventricles. Heart and abdominal ultrasounds were reported normal.

Patient 6 is a 21-month old baby, the second child of non-consanguineous parents. His family history is positive for language delay. He was born at 40 WG by cesarean delivery. He was referred to us for the presence of dysmorphic features and psychomotor delay. On physical examination he had trigonocephaly, epicanthus, down-slanting palpebra fissures, large nasal bridge, thin upper lip, large mouth, small and dysplastic ears, thick fingers and broad thumbs and hallux. Heart and abdominal ultrasounds were reported normal, while brain CT and MRI showed areas of periventricular leukomalacia and mild ptosis of the cerebellar tonsils.

Patient 7 is a 2-year old boy, the first child of non-consanguineous parents, born at 36,1 WG by cesarean delivery. His mother has intellectual disability, thyroid hypoplasia and hypothyroidism, reason why she had performed array-CGH, showing a 1q21.1 deletion.

He has dysmorphic features (sloping forehead, prominent occiput, flat nasal bridge, long philtrum, thin upper lip, large mouth, protruding tongue), congenital hypothyroidism and ectopic urethral meatus. The ultrasound study did not document thyroid hypoplasia. Brain and abdominal ultrasounds were reported normal.

### Array-CGH analysis

Genomic DNA of the patients (except patient 3) and their parents was extracted from peripheral blood lymphocytes using KingFisher Blood DNA Kit (Thermo Scientific, Vantaa, FI) according to manufacturers’ instructions. Proband and reference DNA (Promega Corporation, Madison, WI, USA) were labeled with Cy5-dUTP and Cy3-dUTP respectively. Whole genome array-CGH was performed using Human Genome CGH Microarray Kit 8x60K (Agilent Technologies, Santa Clara, CA, USA) with an average resolution of 100 kb (Build37: Feb 2009-hg19) according to manufacturers’ instructions. Images of the array were acquired with Agilent scanner G2505B and analyzed with Feature Extraction software v9.5.1 (Agilent Technologies, Santa Clara, CA, USA). Graphical overviews of results were obtained with Genomic Workbench Standard Edition software v5.0.14 (Agilent Technologies, Santa Clara, CA, USA).

Genomic DNA of patient 3 was extracted from amniotic cells. Proband and reference DNA (Promega; G147A) were labeled with Cy5-dUTP and Cy3-dUTP respectively. Whole genome array-CGH was performed using CytoChip Oligo arrays 8x60K (Bluegnome, Cambridge, UK) with an average resolution of 200–250 kb. Images of the array were acquired and analyzed with BlueFuse Multi Software for microarray.

## Results

Among our seven patients we detected, using array-CGH, four 1q21.1 deletions, two 1q21.1 duplications and a double rearrangement on the long arm of a chromosome 1, with a 1q21.1 duplication and a 1q21.1-q21.2 deletion.

Before undergoing to array-CGH analysis, a karyotype study was performed in patient 2, 4 and 7: all patients have a normal male karyotype (46,XY).

**Fig. 1:**
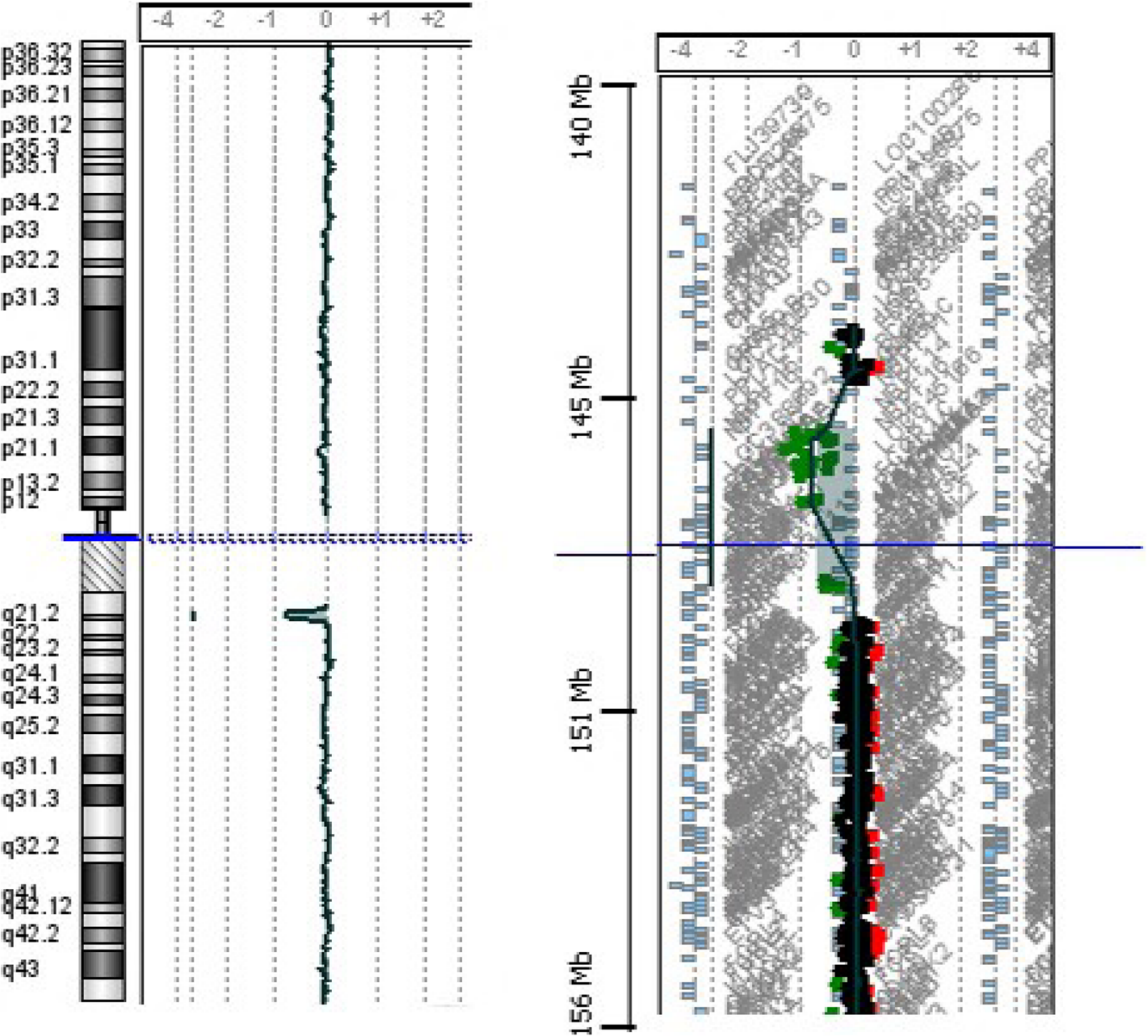
Genome-wide array CGH analysis: arr 1q21.1q21.2(146,641,601–147,786,706)×1, 1,1 Mb deletion of the long arm of chromosome 1, ranging from 146.641.601 Mb to 147.786.706

**Fig. 2:**
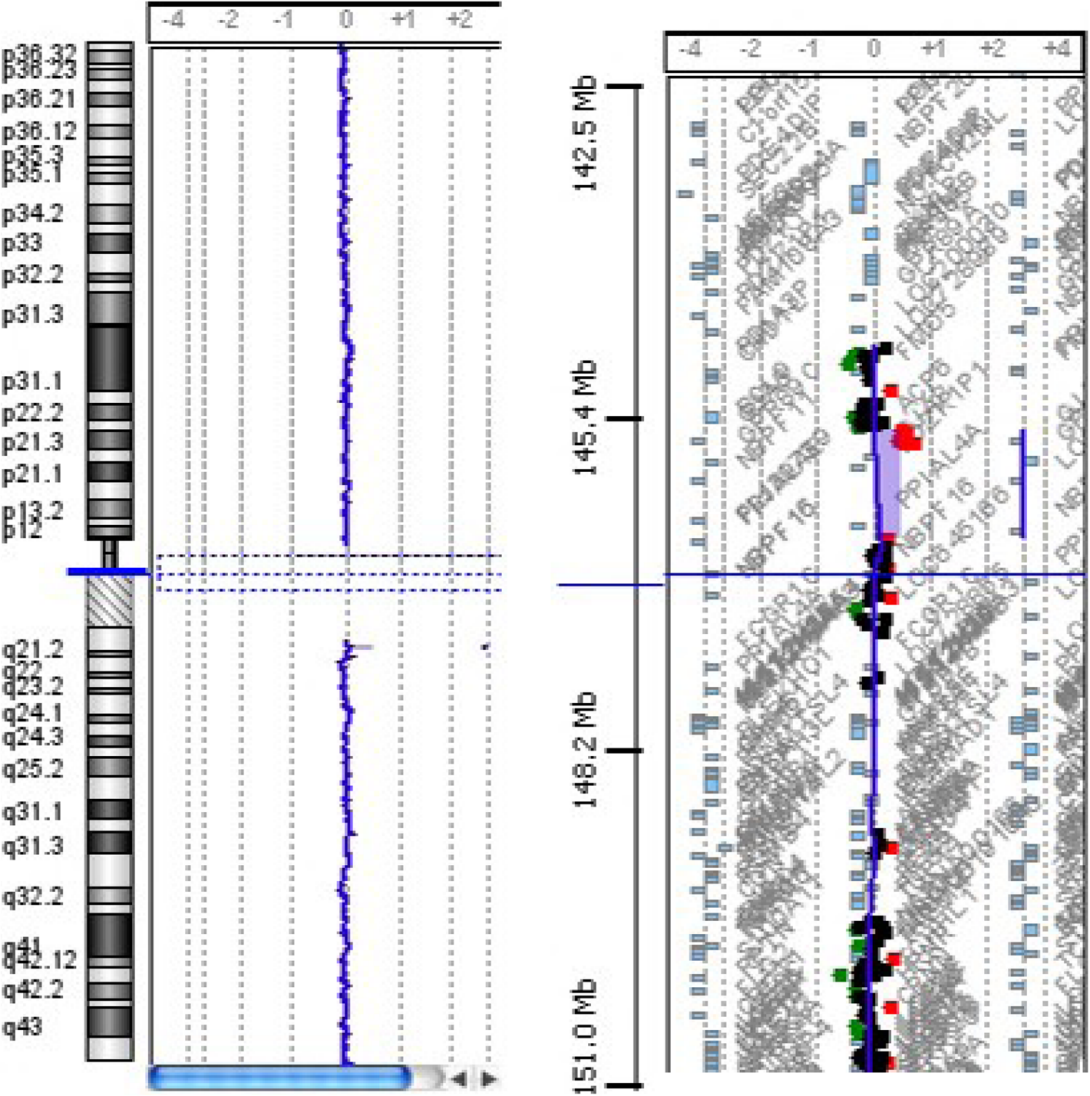
Genome-wide array CGH analysis: arr 1q21.1(145,291,711–145,747,269)×3, 456 kb duplication of the long arm of chromosome 1, ranging from 145.291.711 Mb to 145.747.269 Mb

We detected a 1q21.1 deletion in patient 2, 4, 5 and 7. Patient 2 and 4 have a 1q21.1 deletion that spans approximately 1,2 Mb (146.564.743–147.786.706). Array-CGH analysis in both parents of patient 2 shows that the rearrangement has a paternal origin, while these data are not available for the parents of patient 4. Patient 5 and 7 have a 1q21.1 deletion, of approximately 1,1 Mb (146.641.601–147.786.706)(Fig. 1). The rearrangement is inherited from his mother in patient 7, while it is de novo for what concerns patient 5. This deleted region includes numerous genes: *PRKAB2, PDIA3P, FMO5, CHD1L, BCL9, ACP6, GJA5, GJA8, GPR89B, PDZK1P1, NBPF11, NBPF24*. Patients with a microduplication are patient 1 and 6. Patient 1 has a 1q21.1 duplication of approximately 932 kb (145.632.334–146.564.802), involving the genes: *GPR89A, PDZK1, CD160, RNF115, POLR3C, NUDT17, PIAS3, ANKRD35, PEX11B, ITGA10, RBM8A, LIX1L, POLR3GL, HFE2, NBPF10, NOTCH2NL*. The origin of this rearrangement is unknown, because array-CGH analysis data concerning parents are not available. Patient 6 has a 1q21.1 duplication that spans approximately 456 kb (145.291.711–145.747.269)(Fig. 2). Array-CGH analysis in both parents shows that the rearrangement has a maternal origin. The duplicated region involves genes such as *NBPF20, GPR89A, PDZK1, CD160* and *RNF115*.

Finally, patient 3 is the only one with a prenatal diagnosis. Fetal karyotype on amniotic cells revealed a chromosomal insertion involving the short arm of a chromosome 3 and the short arm of a chromosome 1: 46, XY, ins(1;3)(p22;p13p23). This rearrangement was confirmed by Fluorescent in-situ Hybridization (FISH): ish ins(1;3)(p22;p13p23)(pVYS218C+;wpc3+). The karyotype study in both parents was normal, this showing that the rearrangement was de novo. In order to better define this chromosomal abnormality, array-CGH analysis was performed. We detected a double rearrangement on the long arm of a chromosome 1: a 1q21.1 duplication extended for 1.2 Mb (from 144.612.681 to 145.799.573) and a 1q21.1-q21.2 deletion extended for 1,7 Mb (from 146.155.983 to 147.824.178). These complex rearrangements were confirmed by Dye-swap. Both rearrangements are de novo. The duplicated region includes many genes: *NBPF9, PDE4DIP, NBPF10, HFE2A, TXNIP, RBM8A, PEX11B, ITGA10, PIAS3, CD160, PDZK1, GPR89A*. The 1q21.1-q21.2 deletion comprises the 1q21.1 microdeletion syndrome critical region. This region includes genes such as *PRKAB2, FMO5, CHD1L, BCL9, ACP6, GJA5, GJA8* and *GPR89B*. Though karyotype and FISH analysis performed prenatally revealed a chromosomal insertion involving the short arm of a chromosome 3 and the short arm of a chromosome 1, array-CGH analysis did not show gain or loss of genetic material of these regions. So we can affirm that it is a balanced rearrangement. At the same time, array-CGH allowed us to highlight a double rearrangement involving the long arm of chromosome 1.

(The main characteristics of the patients are shown in Table 1).

**Table 1:** Summary of genotypic and phenotypic characteristics of patients

Patient No.	Dysmorphic features	Cognitive features	Growth features	Skeletal features	Congenital anomalies	Other features	Genotype	Inheritance
1	Mild dismorfic features	Intellectual disability, psychomotor and language delay	Microcephaly; normal height and weight	Normal	-	-	dup1q21.1	Unknown
2	Dry hair with abnormal implant, hypotelorism	Learning disabilities	Height, weight and cranic circunference <3rd percentile	Bilateral clinodactyly of I, II, IV, V fingers	-	Encopresis	del1q21.1	Paternal
3	Normal	Normal	Normal	Normal	-	-	dup1q21.1+ del1q21.1-q21.2	De novo
4	Prominence of the metopic suture, plagiocephaly, hypotelorism	Normal	Height, weight and cranic circunference <3rd percentile	Bilateral clinodactyly of IV and V fingers and toes	Vesicoureteral reflux	-	del1q21.1	Unknown
5	Protruding ears, prominent nasal bridge, short philtrum, micrognathia, spaced teeth	Intellectual disability	Microcephaly; normal height and weight	Normal	-	Spastic tetraparesis, strabism	del1q21.1	De novo
6	Trigonocephaly, epicanthus, down-slanting palpebra fissures, large nasal bridge, thin upper lip, large mouth, small and dysplastic ears	Psychomotor delay	Normal	Thick fingers and broad thumbs and hallux	-	-	dup1q21.1	Maternal
7	Sloping forehead, prominent occiput, flat nasal bridge, long philtrum, thin upper lip, large mouth, protruding tongue	Normal	Normal	Normal	Congenital hypothyroidism, ectopic urethral meatus	-	del1q21.1	Maternal

## Discussion

Recurrent reciprocal 1q21.1 deletions and duplications have been associated with variable phenotypes. The 1q21.1 microdeletion syndrome is characterized by a high variable clinical phenotype. The most common features, even if not constant, are microcephaly, dysmorphism, developmental delay and mild intellectual disability. Several congenital anomalies may be associated with 1q21.1 deletion: CHD, eye abnormalities, skeletal and genitourinary malformations. Psychiatric and behavioral abnormalities, such as ASD, schizophrenia and ADHD, are also described in a minority of patients.

The 1q21.1 reciprocal duplication have been associated with macrocephaly or relative macrocephaly, dysmorphic features, developmental delay, intellectual disability and autism spectrum disorder. Individuals with 1q21.1 CNVs may also have a normal phenotype.

We describe seven patients, who were referred to us for developmental delay/intellectual disability, dysmorphic features and, in some cases, congenital anomalies, in whom we identified by array-CGH analysis: four 1q21.1 deletions, two 1q21.1 duplications and a double rearrangement on the long arm of a chromosome 1, with a 1q21.1 duplication and a 1q21.1-q21.2 deletion. Duplications detected in patient 3 and 6 involve also the proximal part of 1q21.1 region. 1q21.1 proximal microduplications are associated with variable and not defined phenotypes, including intellectual disabiliy, dysmorphic features and behavior problems.

Our data confirm the extreme phenotypic variability associated with 1q21.1 microdeletion and microduplication. We observed common phenotypic features, described in previous studies, such as microcephaly, mild dysmorphic features, developmental delay and intellectual disability. Three patients also have skeletal anomalies (clinodactyly of fingers and toes, broad thumbs and hallux), while two patients have genitourinary malformation (VUR, ectopic urethral meatus). Furthermore, in our cohort of patients we described, for the first time, congenital hypothyroidism in association with 1q21.1 deletion and trigonocephaly associated with 1q21.1 duplication. None of the patients in our study have CHD.

Disagreeing with the literature, one patient harbouring a duplication (patient 1) has microcephaly instead of relative/absolute macrocephaly.

Only one patient (patient 3), the one with a double rearrangement on the long arm of a chromosome 1 prenatally diagnosed, has no phenotypic manifestations: we suggest a careful follow-up in order to evaluate his psychomotor development and the possible occurence of psychiatric and behavioral abnormalities.

Array-CGH analysis in the parents show a parental origin in three patients: two patients inherited the rearrangement from their mother, while one has a paternal inheritance. Among these, only in one case (patient 7) the mother was affected, having intellectual disability, thyroid hypoplasia and hypothyroidism, while the father of patient 2 and the mother of patient 6 have a normal phenotype. In two of our patients the rearrangement occurs de novo; lastly, in two cases data about parents are not available, so the inheritance remains unknown.

**Fig. 3:**
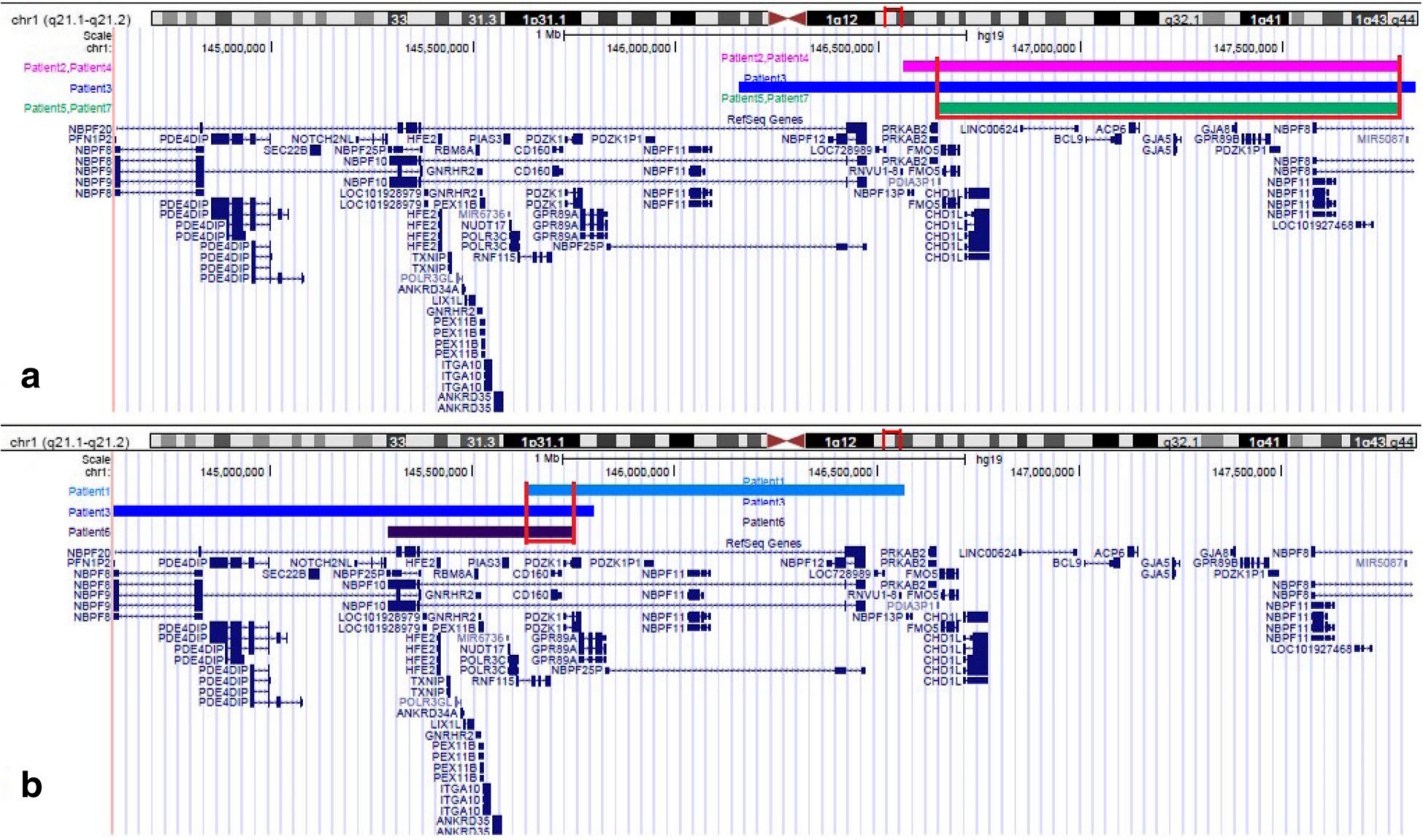
**a.** Mapping of deletion 1q21.1 in patients 2, 3, 4, 5 and 7. The red box indicates the minimal common deleted region. **b.** Mapping of duplication 1q21.1 in patients 1, 3 and 6. The red box indicates the minimal common duplicated region. Note that patient 3 is in both Fig. A and B because of his double rearrangement on the long arm of a chromosome 1

CNVs detected in our patients have different size, ranging from 456 kb to 1,2 Mb (Fig. 3a and b). Many genes are included in the deleted region, but no single gene mutations or haploinsufficiency are known to cause the 1q21.1 microdeletion phenotype. The most studied genes are *CHD1L, PRKAB2, GJA5, GJA8* and *NBPF* genes.

*CHD1L* has been implicated in chromatin remodelling, relaxation and decatenation. Haploinsufficiency or over-expression of *CHD1L* have been implicated in impaired chromatin remodeling during DNA single strand break repair, suggesting that it has a role in DNA Damage Response [10]. Despite that, the phenotypic consequences of alterations in DNA Damage Response in patients with 1q21.1 CNVs are not clear [11].

*PRKAB2* encodes the β2-subunit of AMPK (AMP-activated protein kinase), a regulator of cellular response to a large number of external stimuli, which seems to have an important role in brain function [11].

*GJA5* and *GJA8* have been identified in subjects with cardiac defect and eye abnormalities, respectively [5, 12, 13].

The *NBPF* (neuroblastoma breakpoint family) gene family consists of 24 members. Deleted or duplicated regions involved in our patients include: *NBPF9, NBPF10, NBPF11, NBPF20* and *NBPF24*.

This gene family encodes for the DUF1220 protein domains, that seem to be associated with pathological variations in brain-size and neocortex volume. In particular, the loss of DUF1220 copy number has been associated with microcephaly, while the increases in DUF1220 copy number underlie 1q21-associated macrocephaly [14].

In the duplicated region, the most studied gene is *PDZK1*. Over-expression of this gene has been described in association with an increased risk of ASD and psychiatric diseases [15, 16].

The duplicated region in patient 1 encompasses also the *PEX11B* (peroxisomal membrane protein 11B) gene. It seems to be involved in the regulation of neuronal differentiation and migration, so it could be responsible for pathological variations in brain-size [17].

Among the genes involved in patient 7 (*PRKAB2, PDIA3P, FMO5, CHD1L, BCL9, ACP6, GJA5, GJA8, GPR89B, PDZK1P1, NBPF11, NBPF24*), with current knowledge, none seems to be related with congenital hypothyroidism. We believe it is important to report this finding in order to better define this complex phenotype, although we are aware that more studies are needed to explain the relationship between 1q21.1 deletion and hypothyroidism. Likewise, none of the genes duplicated in patient 6 (*NBPF20, GPR89A, PDZK1, CD160, RNF115*) are known to cause trigonocephaly.

## Conclusions

In conclusion, our results confirm the high phenotypic variability of 1q21.1 deletions and duplications, extending the phenotype with the finding of congenital hypothyroidism and trigonocephaly in association with 1q21.1 deletion and duplication, respectively.

Further studies are needed to better define the 1q21.1 microdeletion/microduplication syndrome and to understand how haploinsufficiency or over-expression of genes included in this region can cause this complex phenotype.

## Abbreviations

ADHD: Attention deficit hyperactivity disorder
ASD: Autism spectrum disorder
CGH: Comparative genomic hybridization
CHD: Congenital heart disease
CNVs: Copy number variations
EEG: Electroencephalogram
FISH: Fluorescent in-situ Hybridization
PROM: Premature rupture of membrans
ROP: Retinopathy of prematurity
VUR: Vesicoureteral reflux
WG: Weeks of gestation
